# Intake of high-fructose corn syrup sweetened soft drinks, fruit drinks and apple juice is associated with prevalent arthritis in US adults, aged 20–30 years

**DOI:** 10.1038/nutd.2016.7

**Published:** 2016-03-07

**Authors:** L R DeChristopher, J Uribarri, K L Tucker

**Affiliations:** 1 Molecular Biology, NY Medical College, Valhalla, NY, USA; 2 Department of Medicine, Icahn School of Medicine, New York, NY, USA; 3 Department of Clinical Laboratory and Nutritional Sciences, University of Massachusetts, Lowell, MA, USA

## Abstract

**Objective:**

There is a link between joint and gut inflammation of unknown etiology in arthritis. Existing research indicates that regular consumption of high-fructose corn syrup sweetened (HFCS) soft drinks, but not diet soft drinks, may be associated with increased risk of seropositive rheumatoid arthritis (RA) in women, independent of other dietary and lifestyle factors. One unexplored hypothesis for this association is that fructose malabsorption, due to regular consumption of excess free fructose (EFF) and HFCS, contributes to fructose reactivity in the gastrointestinal tract and intestinal *in situ* formation of enFruAGEs, which once absorbed, travel beyond the intestinal boundaries to other tissues and promote inflammation. In separate studies, the accumulation of advanced glycation end-products has been associated with joint inflammation in RA. Objective of this study was to assess the association between EFF beverages intake and non-age, non-wear and tear-associated arthritis in US young adults.

**Methods:**

In this cross sectional study of 1209 adults aged 20–30y, (Nutrition and Health Examination Surveys 2003–2006) exposure variables were high EFF beverages, including HFCS sweetened soft drinks, and any combination of HFCS sweetened soft drinks, fruit drinks (FD) and apple juice, referred to as tEFF. Analyses of diet soda and diet FD were included for comparison. The outcome was self-reported arthritis. Rao Scott Ҳ^2^ was used for prevalence differences and logistic regression for associations, adjusted for confounders.

**Results:**

Young adults consuming any combination of high EFF beverages (tEFF) ⩾5 times/week (but not diet soda) were three times as likely to have arthritis as non/low consumers (odds ratios=3.01; *p*⩽0.021; 95% confidence intervals=1.20–7.59), independent of all covariates, including physical activity, other dietary factors, blood glucose and smoking.

**Conclusion:**

EFF beverage intake is significantly associated with arthritis in US adults aged 20–30 years, possibly due to the intestinal *in situ* formation of enFruAGEs.

## Introduction

Arthritis encompasses musculoskeletal disorders consisting of >100 different conditions that destroy joints, bones, muscles, cartilage and other connective tissues, hampering or halting physical movement. With the exception of ‘wear and tear' age-associated osteoarthritis, most forms of arthritis are auto-immune disorders that are characterized by inflammation wherein the exact etiologies are unknown.^1^ According to the US Center for Disease Control, during the period of this study, 21.6% of the adult US population (46.4 million persons) had doctor-diagnosed arthritis and 8.3% (17.4 million) had arthritis-attributable activity limitations. Arthritis is the leading cause of disability in the US.^2^

Although the risk of developing most types of arthritis increases with age, arthritis also occurs in young people. Types of arthritis most known to affect young adults include ankylosing spondylitis,^3^ rheumatoid arthritis (RA),^4, 5^ lupus erythematosus,^6, 7^ psoriatic arthritis^8^ and undifferentiated arthritis.^9, 10^

Notably, in many arthritis patients there is a link between joint and gut inflammation. For example, gastrointestinal disease is one of the most common comorbidities in patients with RA^11, 12^ and lupus erythematosus.^13^ Coincident gut and joint inflammation is also common in ankylosing spondylitis and psoriatic arthritis.^14^ While adverse gastrointestinal symptoms are known side effects of glucocorticoids and excessive nonsteroidal anti-inflammatory drug use, existing research provides evidence that gastrointestinal symptoms of unknown etiology are independent of medication use in a subset of patients. There is evidence that there may be an immunological link between gut immunity and arthritis of dietary origin.^15, 16, 17, 18^ Some researchers have suggested that gut bacteria may be involved in the coincident gut and joint inflammation, but the ‘gut bacteria model' does not explain the co-localization of inflammation to the synovium and gut, or identify the specific bacterial antigens that may incite inflammation.^14^

Interestingly, a recent large scale prospective epidemiology study of nearly 200,000 women (Nurses Health Study) found that regular consumption of high-fructose corn syrup (HFCS) sweetened soft drinks, but not diet soft drinks, was associated with increased risk of seropositive RA in adult women, independent of weight and lifestyle factors. Researchers concluded that the deleterious effect of non-diet soda on RA development might not be through weight gain and obesity, as the risk of RA was not attenuated after adjustment for weight change and body mass index (BMI). Researchers suggested that non-diet soda intake might increase the risk of RA by upregulating inflammatory cytokines and decreasing insulin sensitivity due to the glycemic load of non-diet soft drinks (ndSD), and possibly due to the advanced glycosylation (AGEs) end-products present in the caramel coloring of soda.^4^

However, this hypothesis is unclear for three reasons. First, existing research indicates that ndSD are not a significant source of AGEs.^19^ Second, diet soft drinks contain the same caramel coloring as ndSD, but were not found to be associated with RA.^4^ Third, their results were independent of diabetes history,^4^ suggesting that mechanisms other than glycemic load may underlie the ndSD association with RA. Notably, HFCS, and not sucrose, is the main sweetener is US soft drinks.

Another unresearched and overlooked source of AGEs that may be arthrogenic is intestinally generated AGEs. According to the intestinal enFruAGE ‘fructositis' hypothesis, underlying fructose malabsorption (FM) and unabsorbed EFF intake, contributes to the intestinal *in situ* formation of advanced glycation end-products (enFruAGEs) that may be an overlooked source of pro-inflammatory AGEs that travel beyond the intestinal boundaries to other tissues and may play a role in the etiology of auto-immune arthritis.^20, 21^ It is noteworthy that adult FM is not associated with consumption of sucrose or equal monomers of fructose and glucose.^22, 23, 24, 25, 26, 27^

It is possible that enFruAGEs may play a role in idiopathic arthritis, as existing research provides evidence that AGEs are elevated in connective tissues including the synovium, sub-lining and cartilage of arthritis sufferers.^28, 29, 30, 31, 32^ This possibility is increased because of evidence that more EFF is being consumed than has been assumed; independent lab results indicate that popular soda beverages are sweetened with an HFCS variant that is 60% fructose and 40% glucose, rather than the 55/45 formula that is generally recognized as safe.^33, 34, 35^ Different varieties of fruit drinks (FD) with high apple juice (AJ) content were found to contain a 67/33% combination of fructose and glucose monomers.^33, 34, 35^ This is because AJ, unlike orange juice, is also a high-excess-free fructose beverage.^36^

This study, examined a potential role for enFruAGEs in arthritis pathogenesis by studying the association between young adult arthritis prevalence and intake frequency of high EFF beverages, including HFCS sweetened soft drinks,^36^ FD and AJ^36^ in a large database containing information about beverages intake and arthritis prevalence in adults ages 20 and older (Nutrition and Health Examination Surveys (NHANES) 2003–2006). Analysis of diet soda (a non-EFF beverage) was conducted for comparison. For this analysis the focus was on young adults, aged 20–30 year, as the interest was in a cohort more likely to be diagnosed with an autoimmune variant of arthritis and less likely to be diagnosed with ‘wear and tear' age-associated osteoarthritis. In addition, young adults consume significantly more ndSD than older adults.^37^ There were 1084 study participants.

## Methods

### Sample and survey administration

Cross-sectional data were used to assess the association between high EFF beverages intake and arthritis using a nationally representative survey. NHANES are administered by the US Center for Disease Control and are part of their ongoing mission to produce vital and health statistics for the US.^38^ NHANES for the years of this analysis included a food frequency questionnaire (FFQ). A consistent relationship between reported FFQ frequency of food and food-group consumption and probability of consumption on 24-h recalls has been demonstrated.^39^ Data from FFQ are used as a reliable measure of dietary patterns and food intake information in epidemiological research.^4, 40, 41^

NHANES for the years 2003 through 2006 included an FFQ with frequency of intake for the beverages of interest as potential predictor variables. The medical questionnaire component included arthritis status information. Because NHANES uses a complex sampling design and constructs sample weights to produce nationally representative data, sample weights provided in the food frequency data files were used to perform statistical analysis for this study.^38^

The weights account for oversampling of various groups and reflect the fact that, of the 20,470 people who participated in the overall (4 year) study, not all respondents (11,505) participated in the food frequency part of the survey.^42^ Therefore all statistics and summary tables are appropriately weighted.

### Variables

The outcome variable was self-reported arthritis or history of arthritis. On the NHANES questionnaire, this was asked as ‘Has a doctor or other health professional ever told you that you have arthritis?' In the NHANES, the target age group for this question was adults, aged 20 and older. Beverage intake exposure variables were obtained from the FFQ questions: How often did you drink (1) AJ? (2) orange or grapefruit juice? (3) other FD (such as cranberry cocktail, lemonade and so on) (4) soft drinks, soda or pop in the summer? and (5) soft drinks, soda or pop the rest of the year? Additional questions clarified how often FD or soft drinks were diet or sugar-free or caffeine free.^38^

The average daily frequency of ndSD and diet soft drinks over the past year was calculated by summing individual values for caffeinated and caffeine-free non-diet soda-in-the-summer and rest-of-year. The NHANES utilized specialized software (Diet-Calc) to assign frequencies to responses from the FFQ using algorithms as follows: never=0; 1 time per month or less=0.03; 2–3 times per month=0.08; 1–2 times per week=0.21; 3–4 times per week=0.5; 5–6 times per week=0.79; 1 time per day=1; 2–3 times per day=2.5.^38^ Intake data were combined to establish new intervals for analysis purposes as follows: ⩽2–3 times per month as the reference group; 1–4 times per week and ⩾5 times per week.

To analyze the combined effects of EFF, the following algorithm was used. Intake frequencies for ndSD, FD and AJ were assigned a zero for once a month or less; 0.117 for 2–3 times a month; 0.357 for 1–4 times per week and 1 for >5 times per week. These values were summed to calculate average daily intake of total EFF beverages. The same approach was used to analyze the combined effects of diet soft drinks and diet FD.

Adjustment variables included sex, race/ethnicity, age, BMI, total energy intake, smoking, family income, head of household education level, physical activity, total fruits and vegetables intake, a measure often used as a barometer of healthy lifestyle, and glycated hemoglobin (A1c), a measure of the average level of blood glucose over the previous three months used in the diagnosis and assessment of control of diabetes.^38^ Adjustment variables were selected for use in this study based on existing research.^4, 40^ Total energy intake and total fruit and vegetable intake were the only variables obtained from the 24-h dietary recall and were calculated using either the average of two 24-h recalls or one 24-h recall if the second recall was missing.

Smoking and history of smoking was self-reported. This was asked as a series of questions including, ‘Do you now smoke cigarettes? During the past five days, did you use cigarettes?' Have you smoked at least 100 cigarettes in your entire life? For analysis purposes these were used to distinguish between non-smokers, non-smokers with a history of smoking and current smokers.^38^

Socioeconomic status (SES) was included as a potential confounding variable using data obtained for family income and the head of household education level. NHANES used the Family Interview Income Questionnaire to obtain combined family income for 13 income ranges.^38^ For analysis purposes these were reduced to 0–$19,999; $20,000–$34,999; $35,000–$54,999; $55,000 and over. Head of household education level was obtained by asking, ‘What is the highest grade or school level you have or the head of household has received?' Categories were <9th grade; 9th–11th grade; HS/ GED; some college; and college graduate.^38^ For analysis purposes these were reduced to HS/GED and below, and some college and above.

The NHANES asked several questions to obtain information of physical activity and provided recommended metabolic equivalent scores for each activity. For analysis purposes, metabolic equivalent scores were summed and divided into three equal quantiles. The physical activity questions and corresponding metabolic equivalent scores used in this study are as follows: which of four sentences best describes your usual daily activities? You sit during the day and do not walk about very much (1.4). You stand or walk about a lot during the day, but do not have to carry or lift things very often (1.5). You lift light loads or climb stairs or hills often (1.6). You do heavy work or carry heavy loads (1.8). Have you over the past 30 days, walked or bicycled to get to/from work (4.0); done tasks in or around home or yard that required moderate or greater physical effort (4.5); done moderate activities that caused light sweating or moderate increases in breathing or heart rate including brisk walking, bicycling for pleasure, golf, and dancing, and so on (3.5); done vigorous activities that caused heavy sweating or large increases in breathing or heart rate including exercise, sports and other active hobbies (7.0).^38^

### Statistical analysis

Analysis was performed utilizing statistical software from STATA Corporation (College Station, TX, USA), revision 18. Appropriate procedures were used to account for complex sample design. As previously described, weight variables were used to account for non-response and oversampling of various age groups and ethnic groups. Rao Scott Ҳ^2^ analysis was used to test for significance of differences in arthritis prevalence by intake frequency. Analysis was conducted individually for intake frequencies of diet soft drinks; HFCS soft drinks; any combination of HFCS sweetened soft drinks, FD and AJ (tEFF); and any combination of diet soft drinks and diet FD. A *p*-value of ⩽0.05 was considered significant, with values <0.10 approaching significance.

Logistic regression was used to assess the adjusted odds between exposure variables and arthritis, independent of confounding variables, for the same beverages and beverage groups analyzed using Rao Scott Ҳ^2^. Two multivariate logistic regression models were used to analyze adjusted odds ratios (OR) as follows: the first model adjusted for age, sex, race/ ethnicity, BMI, SES, total energy intake, total fruit and vegetable intake, physical activity level, and smoking. The second model also adjusted for glycated hemoglobin (A1c) – a measure of the average level of blood glucose over the previous three months. During individual analysis of ndSD, an adjustment for AJ and FD was done to assess the association between HFCS sweetened soft drinks and arthritis, independent of the other high EFF beverages. In logistic regression analysis, confidence intervals (CI) that did not include 1 and *p*-values ⩽0.05 were considered statistically significant.

## Results

Consumption of beverages high in excess-free fructose was significantly correlated with arthritis in US young adults aged 20–30 years. Overall, 4.6% of 1084 adults, aged 20–30 years were reported to have arthritis (or history of arthritis). A comparison of arthritis prevalence with national statistics was not possible for this age sub-group, as available US Centers for Disease Control and prevention data only include adults aged 18–44 years (7.3%).^2^ However, analyses based on NHANES are generalizable to the US non-institutionalized civilian population.^38^ Descriptive characteristics of the sample are presented in Table 1. There was a statistically significant association between increasing intake of any combination of HFCS sweetened soft drinks, FD and AJ and increased odds of arthritis in this age group (*p*⩽0.05), but not with diet drinks.^43, 44^

Unadjusted Rao Scott Ҳ^2^ comparisons with arthritis prevalence showed that intake of non-diet soda and any combination of high EFF beverages (tEFF) was highly significant, but not intake of diet soda and diet FD. Arthritis prevalence was significantly higher among young adults who regularly consumed ndSD (3.8%), or any combination of high EFF beverages (non-diet soda, AJ and FD) (3.5%), relative to non/low consumers (0.7%) and (0.5%). Table 2. Arthritis prevalence among young adults consuming non-diet soda ⩾5 times per week (3.8%) and tEFF ⩾5 times per week (3.5%) was six and five times that of non/ low consumers, *p*=0.0021, *p*=0.0133. Arthritis prevalence was low among regular consumers of diet soft drinks and diet FD (1.1%), relative to non/ low consumers (0%), *p*=0.2159. Table 2.

**Figure 1:**
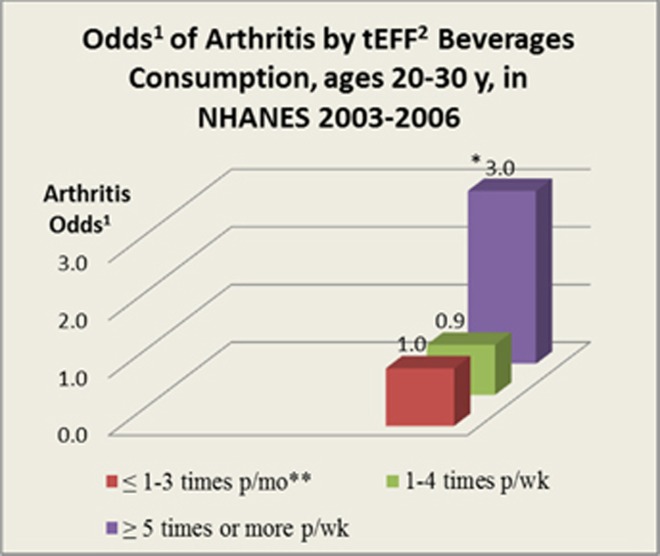
*OR=3.01, *p*=0.021, 95% CI=1.18–7.25, *N*=1046; **⩽1–3 X's per month is the reference group. (1) Odds of arthritis adjusted for age, sex, race, BMI, total energy intake, total fruit and vegetable intake, socio-economic status, physical activity, smoking, and glycated hemoglobin. (2) tEFF refers to total excess free fructose, which is any combination of ndSD, FD and AJ (high EFF beverages).^36^

In logistic regression models adjusted for age, sex, race/ ethnicity, BMI, SES, physical activity, total energy intake, total fruit and vegetable intake (a barometer of healthy lifestyle), smoking, history of smoking, and glycated hemoglobin (A1c), the cumulative effect of HFCS sweetened soft drinks, FD, and AJ (tEFF) was significant. Young adults reporting tEFF intake ⩾5 times per week had three times higher odds of arthritis as ⩽1–3 times per month consumers, after adjusting for potentially confounding variables (OR=3.01; *p*⩽0.021; 95% CI=1.20–7.59). Figure 1. Although there was a tendency toward greater risk, individual consumption of ndSD was not significantly associated with arthritis after adjustment for potential confounders (OR=2.68; *p*⩽0.102; 95% CI=0.81–8.84). Table 3.

In logistic regression analysis of diet beverages, the reference group was set to ⩽1–4 times per week as there was no arthritis prevalence among non/ low (⩽1–3 times per month) diet drink consumers. After adjustment for all potential confounders, consumption of diet soft drinks ⩾5 times per week was not associated with arthritis (OR=1.26; *p*⩽0.797; 95% CI=0.20–7.93), nor was any combination of diet soft drinks and diet FD (OR=1.21; *p*⩽0.759; 95% CI=0.35–4.23) Table 3.

## Discussion

Consumption of a combination of HFCS sweetened soft drinks, FD and AJ (tEFF) was significantly associated with arthritis in young adults aged 20–30 years, independent of known co-morbidities, including other dietary lifestyle factors, physical activity, BMI, smoking, and glycated hemoglobin concentration (OR=3.01; *p*⩽0.021; 95% CI=1.20–7.59), but there was no association with diet drinks (OR=1.21; *p*⩽0.759; 95% CI=0.35– 4.23).

These results provide support for the possibility that the correlation with young adult arthritis is due to the excess free fructose in HFCS sweetened soft drinks, FD and AJ. Results are consistent with the possibility that underlying fructose malabsorption and unabsorbed EFF, contributes to the intestinal in situ formation of advanced glycation end-products (enFruAGEs) that may be an overlooked source of pro-inflammatory AGEs that travel beyond the intestinal boundaries to sites of arthritis tissue inflammation.^21^ Notably, existing murine-based research provides evidence of AGEs formation in the jejunum.^45, 46^

Arthritis prevalence among regular consumers (⩾5 times per week) of tEFF beverages (3.5%) and HFCS sweetened soft drinks (3.8%) was significantly higher than among non/low consumers (0.5/0.7%). In comparison, arthritis prevalence among regular consumers of diet soft drinks and diet FD (1.1%) was considerably lower than arthritis prevalence among regular consumers of HFCS sweetened soft drinks (3.5%). This difference provides further evidence and support for the hypothesis that it may be the cumulative effect of excess free fructose that is underlying the association with young adult arthritis.

Existing FM research provides insights into how beverages with similar levels of total sugars, but different fructose composition could have different effects on the body. Research indicates that adult FM occurs after consumption of EFF, but not after consumption of sucrose or equal amounts of fructose and glucose monomers,^22, 23, 24, 25, 26, 27^ possibly because they are transported and absorbed differently by the body during digestion.^47^ An analysis of the sugar composition of HFCS sweetened soft drinks, AJ and orange juice helps to illustrate how HFCS sweetened soft drinks and FD have more in common with AJ, than AJ has with orange juice.

According to the USDA National Nutrient Database for Standard Reference (NDB) release 26 per 100 g, the amount of total sugars in AJ and orange juice are comparable; AJ contains 9.6 g and orange juice contains 8.8 g of total sugars.^36^ Similarly, the total fructose amounts in AJ and orange juice are comparable; per 100 g, AJ contains 6.4 g and orange juice contains 4.5 g of fructose.^36^ Whereas analysis of their EFF content indicates that these juices are quite different from one another. Specifically, the excess free fructose content in an 8 oz cup of AJ is 7.7 g (NDB no. 09400) and in orange juice it is 0.4 g (NDB no. 09207). Per (8 oz.) cup, AJ contains 19 times more EFF than orange juice.^36^ Therefore, AJ is a high EFF beverage, whereas orange juice is not.

Although AJ is a higher EFF beverage than HFCS sweetened soft drinks, and contributes to total excess free fructose load,^36^ in the US, HFCS sweetened soft drinks are the most significant source of EFF in adult diets,^37, 48, 49^ even though many other foods are sweetened with HFCS.^48, 49^ Present estimates indicate that the US average per capita consumption of HFCS is just under a lb. per week, or 65 grams per day,^48^ which is the same amount of HFCS in a 20 ounce bottle of non-diet cola.^50^ When sweetened with the 60/40 variant of HFCS, a 20 ounce bottle of cola contains 13 g of EFF. Therefore, EFF intakes may exceed dosages associated with adult FM in subsets of the population, as FM research indicates that 30% of healthy adults are FM positive after a 25 g EFF dose, and 10% are FM positive after a 12 g EFF dose, but not after consuming sucrose or equal amounts of fructose and glucose monomers.^22^

It is also noteworthy that regular consumption of tEFF beverages remained significantly associated with young adult arthritis even after adjustment for glycated hemoglobin concentration. This is consistent with research of glycation in arthritis, wherein early onset arthritis patients with elevated IgG-AGE had significantly higher concentrations of the inflammatory markers C-reactive protein and erythrocyte sedimentation rate, but there was no correlation with blood glucose.^51^ As there was no correlation between IgG-AGE and glucose, researchers hypothesized that it was the inflammation and not hyperglycemia that exerts the major influence on AGEs formation in patients with early synovitis.^51^ This supports the possibility that intestinally formed enFruAGEs, due to underlying fructose malabsorption, may contribute to arthritis-associated inflammation.

This conclusion is further supported by the 2014 prospective epidemiologic study of nearly 200,000 women (Nurses Health Studies), which found that the association between regular consumption of ndSD and seropositive rheumatoid arthritis in women (but not with diet soda) was independent of diabetes history.^4^ This suggests that the link with RA may be driven, at least in part, by factors other than glycemic load, β cell malfunction, and insulin insensitivity. This largescale longitudinal study provides further evidence that excess free fructose in HFCS sweetened soft drinks may be responsible for the statistically significant association between regular consumption of ndSD and rheumatoid arthritis in women.

This study's results and the results of the 2014 Nurses Health Studies should be considered in the context of research that has focused on glycation in arthritis. By-products of glycation that have been identified in synovial tissues, sub-lining and endothelium of arthritis patients correlate with inflammation.^28, 29, 30, 31, 32^ Connective tissues including synovial tissues and cartilage contain relatively high levels of membrane bound receptor of advanced glycation end-products (RAGE) that are associated with pro-inflammatory signaling.^52^ Existing research provides evidence that RAGE is overexpressed in tissues with increased ligands of RAGE.^53^ RAGE overexpression has been implicated in the pathogenesis of arthritis.^53, 54^ In contrast, soluble RAGE (sRAGE) acts as a decoy receptor and down regulates inflammation. Decreased sRAGE is associated with increased arthritis prevalence.^55, 56^

These results should also be considered in the context of research that has provided evidence that lymphocytes and macrophages traffic from the gut to synovial tissues, but the inciting antigen or [gut] immune trigger has remained unclear.^14^ It should be considered along with research that has provided evidence of inflammatory bowel disease, or early Crohn's disease, in the majority of ankylosing spondylitis patients evaluated, regardless of genetic marker HLAB27.^57^ Additional consideration should be given to research indicating that there may be an immunological link between gut immunity and arthritis of dietary origin.^15, 16, 17, 18^ The link between regular consumption of high EFF beverages and arthritis prevalence may explain, at least in part, the unexplained gut and joint inflammation prevalence in idiopathic arthritis.

Interestingly, results of a large scale longitudinal study (the European Prospective Investigation of Cancer in Norfolk (EPICNorfolk)) provide support for a role for AGEs in polyarthritis. After adjusting for total energy intake, smoking, and other possible dietary confounders, participants with the highest intake of red meat (OR=1.9, 95% CI=0.9-4.0), meat and meat products combined (OR=2.3, 95% CI=1.1–4.9), or total protein (OR=2.9, 95% CI=1.1–7.5) were at an increased risk for inflammatory polyarthritis.^16^ It is possible that dietary AGEs play a role in the association between red meat intake and arthritis, as cooked red meat contains high levels of dietary AGEs, relative to other foods.^19^

This study's results, along with existing research, provide support for the hypothesis that gastrointestinal generated enFruAGEs may be inciting antigens and gut immune triggers that cause lymphocytes and macrophages to traffic from the gut to synovial tissues.^14^ However, the gut immune trigger has remained elusive. EnFruAGEs could account for^21^ the postulated relationship between observed intestinal abnormalities and the pathogenesis of some forms of arthritis, independent of glucocorticoids and nonsteroidal anti-inflammatory drug use.^15, 16, 17, 18^

Lastly, these results should also be considered in the context of recently published epidemiologic studies which indicate that excess free fructose intake is associated with other pro-inflammatory diseases known to be associated with elevated levels of advanced glycation end-products and the RAGE including childhood asthma,^20, 41, 58, 59, 60^ and adult chronic bronchitis, a subcomponent of COPD.^61^ It is noteworthy that chronic respiratory disease is a common co-morbidity of arthritis, as 19% of people with arthritis report a history of chronic respiratory conditions, according to the US Centers for Disease Control and Prevention.^2^

This study is subject to limitations. First, the associations are cross-sectional; therefore, the exposures and outcome are simultaneously assessed. For this reason, cross-sectional studies do not provide evidence of a temporal relationship between exposures and outcome. Follow-up longitudinal studies are needed. Second, it is possible that an association between EFF beverages consumption and arthritis exists in teens. However, this could not be assessed, as in the NHANES, the target age group for questions of arthritis status was adults ages 20 and older. Therefore, analysis of teens aged 15–19 – a group that consumes more ndSD than other age groups^62^ - was not possible, as this data was unavailable. Third, it is possible that in adults over age 30, there is an association between EFF beverages consumption and autoimmune arthritis. However, this was not assessed, as in the NHANES, unlike the Nurses Health Study, there were no questions which sought to distinguish between types of arthritis. Therefore, in older adults, analysis of autoimmune arthritis, as distinguished from wear and tear, age associated osteoarthritis was not possible. Fourth, arthritis status in NHANES is based on self-report, so there is potential for reporting bias. Fifth, high EFF beverages are only one food category that could contribute to daily EFF load. Numerous other food categories contain HFCS as an added sweetener, and therefore, also contribute to daily EFF load. However, among US adults, particularly young adults, soft drinks are the most significant source of HFCS in the American diet.^37, 63^ Sixth, it is possible that the prevalence of arthritis is not raised among high EFF beverages consumers, but is instead reduced among non/low consumers due to unknown lifestyle or protective factors that may be conferred by diets low in EFF beverages, although our results remained significant after adjusting for lifestyle and other dietary factors.

## Conclusion

Regular consumption of high EFF beverages is associated with young adult arthritis. Results support the possibility that there exists a nexus between fructose malabsorption, regular EFF consumption, and increased odds and arthritis prevalence in US adults aged 20–30 years. Unabsorbed excess free fructose may interact with dietary proteins resulting in the in situ formation of enFruAGEs that may play a role in the idiopathic link between gut and joint inflammation. Study results are consistent with the possibility that enFruAGEs are an overlooked source of AGEs that may contribute to arthritis pathogenesis in young adults. Longitudinal and biochemical research are needed to confirm and clarify the mechanisms involved.

## Figures and Tables

**Table 1:** Characteristics of young adults, aged 20–30 years in the NHANES 2003–2006

Number of subjects (N)	1084
Age (year, mean±s.d.)	25±2.6
Sex (%, male)	45.6
	
*Race/ethnicity (%)*	
NHW	66.6
NHB	12.1
Mexican Am	12.4
Other Hisp	2.9
Other	5.9
	
BMI (mean±s.d.)	27.2±6.1
Energy intake (kcal, mean±s.d.)	2350±771
Fruit and vegetable intake (kcal, mean±s.d.)	847±920
	
*Arthritis (%)*	
No	95.4
Yes	4.6

Abbreviations: NHB, non-hispanic black; NHW, non-hispanic white.

**Table 2:** Crude associations between beverage intakes and arthritis in young adults aged 20–30 years in the NHANES 2003–2006

	n	*Proportion* *%*	*95% CI*	*Arthritis (%, yes)*	p*-value*
a*tEFF (AJ, FD, ndSD)*	1084				
⩽1–3 times per month		18.20	14.7–22.4	0.50	0.0133
1-4 times per week		29.30	25.5–33.5	0.50	
5 times or more per week		52.50	47.7–57.1	3.50	
					
b*Non-diet soft drinks (ndSD)*	954				
⩽1–3 times per month		21.60	18.1–25.4	0.70	0.0021
1–4 times per week		31.90	28.0–36.2	0.40	
5 times or more per week		46.50	41.8–51.2	3.80	
					
c*Diet soft drinks*	319				
⩽1–3 times per month		20.50	14.9–27.5	0.00	0.3568
1–4 times per week		39.70	31.5–48.6	1.00	
5 times or more per week		39.70	31.7–48.4	1.10	
					
d*Diet soft drinks+diet FD*	393				
⩽1–3 times per month		21.30	15.5–28.7	0.00	0.2159
1–4 times per week		38.60	31.7–46.0	1.00	
5 times or more per week		40.00	32.8–47.6	1.10	

a tEFF refers to any combination of excess free fructose beverages including ndSD, AJ and FD intake in the NHANES for the period of 2003–2006.^38^

b ndSD corresponds to average daily intake of caffeinated and caffeine free, non-diet soda.^38^ In 2003–2006 (the NHANES study period) HFCS was the main sweetener in soda.^49^

c Diet soft drinks corresponds to average daily intake of caffeinated and caffeine free, diet soda.^38^

d Diet soft drinks and FD correspond to any combination of diet soda and diet FD.^38^

**Table 3:** Associations between excess-free-fructose beverages and arthritis in young adults, aged 20–30 years – NHANES 2003–2006

*Arthritis*	*Multivariable logistic regression*			*Multivariable logistic regression*		
	*ratio*	*95% CI*	p*-value*	*ratio*	*95% CI*	p*-value*
	**OR –** adjusted for sex, race, age,BMI, SES, total energy intake,total fruit and vegetable intake,and physical activity level, smoking			**OR** – further adjusted for glycated hemoglobin (A1c)		
***tEFF (ndSD, FD, AJ)***^a^						
~⩽1–3 times per month	Reference	Reference	Reference	See Figure 1		
~1–4 times per week	0.86	0.21–3.48	0.827			
5 times or more per week	**3.1**	**1.28–7.51**	**0.014**			
* n*=1084	*F*(18, 13)=3.32 Prob>*F*=0.0161					
	**OR –** adjusted for sex, race, age,BMI, SES, total energy, total fruit and vegetable, physical activity level, AJ *and* FD intake, and smoking			**OR** – further adjusted for glycated hemoglobin (A1c)		
						
***ndSD***						
⩽1–3 times per month	Reference	Reference	Reference	Reference	Reference	Reference
1–4 times per week	0.46	0.11–1.84	0.259	0.35	0.07–1.74	0.193
5 times or more per week	2.68	0.81–8.84	0.102	2.56	0.72–9.09	0.14
* n*=954	*F*(22, 9)=6.71 Prob>*F*=0.0029			*F*(23, 8)=8.11 *n*=919 Prob>*F*=0.0072		
	**OR –** adjusted for sex, race, age,BMI, SES, total energy, total fruit and vegetable, physicalactivity level, and smoking			**OR** – further adjusted for glycated hemoglobin (A1c)		
						
***Diet soft drinks***						
⩽1–4 times per week^b^	Reference	Reference	Reference	Reference	Reference	Reference
5 times or more per week	1.13	0.17–7.58	0.898	1.26	0.20–7.93	0.797
* n*=319	*F*(15, 16)=9.31 Prob>*F*=0.0000			*F*(16, 15)=5.21 *n*=310 Prob>*F*=0.0013		
	**OR –** adjusted for sex, race, age,BMI, SES, total energy, total fruit and vegetable, physical activity level, and smoking			**OR** – further adjusted for glycated hemoglobin (A1c)		
						
***Diet soft drinks, diet*** FD						
⩽1–4 times per week^b^	Reference	Reference	Reference	Reference	Reference	Reference
5 times or more per week	1.15	0.31–4.19	0.827	1.21	0.35–4.23	0.759
*n*=379	*F*(15, 16)=6.82 Prob>*F*=0.0002			*F*(16, 15)=4.96 *n*=364 Prob>*F*=0.0017		

a tEFF refers to any combination of excess free fructose beverages including non-diet soda (caffeinated and decaf), AJ and FD intake in the NHANES for the period of 2003–2006.^38^ ndSD corresponds to average daily intake of caffeinated and caffeine free, non-diet soda. In 2003–2006 (the NHANES study period) HFCS was the main sweetener in soda.^49^ AJ is one of very few foods known to contain fructose in high relative proportion to glucose – ~2.9–1.^36^ FD corresponds to sweetened fruit beverages other than juices. Examples given were of beverages known to contain HFCS as the main sweetener.^38^

b As there was zero arthritis prevalence among ⩽1–3 times per month diet soda and diet FD consumers, the reference group for diet beverages was set to ⩽1–4 times per week. See Table 2 for details of arthritis prevalence by beverage type.
